# Solvent and Intermediate Phase as Boosters for the Perovskite Transformation and Solar Cell Performance

**DOI:** 10.1038/srep25648

**Published:** 2016-05-09

**Authors:** Jinhyun Kim, Taehyun Hwang, Sangheon Lee, Byungho Lee, Jaewon Kim, Gil Su Jang, Seunghoon Nam, Byungwoo Park

**Affiliations:** 1 WCU Hybrid Materials Program, Department of Materials Science and Engineering, Research Institute of Advanced Materials, Seoul National University, Seoul 08826, Korea; 2 Nano Mechanical Systems Research Division, Department of Nano Mechanics, Korea Institute of Machinery & Materials (KIMM), Daejeon 305-343, Korea

## Abstract

High power conversion efficiency and device stabilization are two major challenges for CH_3_NH_3_PbI_3_ (MAPbI_3_) perovskite solar cells to be commercialized. Herein, we demonstrate a diffusion-engineered perovskite synthesis method using MAI/ethanol dipping, and compared it to the conventional synthesis method from MAI/iso-propanol. Diffusion of MAI/C_2_H_5_OH into the PbCl_2_ film was observed to be more favorable than that of MAI/C_3_H_7_OH. Facile perovskite conversion from ethanol and highly-crystalline MAPbI_3_ with minimized impurities boosted the efficiency from 5.86% to 9.51%. Additionally, we further identified the intermediates and thereby the reaction mechanisms of PbCl_2_ converting into MAPbI_3_. Through straightforward engineering to enhance the surface morphology as well as the crystallinity of the perovskite with even faster conversion, an initial power conversion efficiency of 11.23% was obtained, in addition to superior stability after 30 days under an ambient condition.

The feasible challenges in solar cell commercialization are enhancement in power conversion efficiency (PCE) and cost reduction to support the world-wide electricity consumption^1^,^2^,^3^,^4^,^5^. Alternatively, organometallic perovskite solar cells were first demonstrated by Miyasaka’s group in 2009 with a PCE of 3.8%^6^, and an enormous growth has been achieved over the last 6 years with the highest efficiency of 22.10%^7^. Perovskite (CH_3_NH_3_PbI_3_) solar cells are settled as the most attractive topic in photovoltaic research areas due to the low fabrication cost and high efficiencies, followed by inherent advantages of the perovskite material which include an appropriate and direct bandgap, small exciton binding energy, balanced ambipolar charge transport properties, etc.^8^,^9^,^10^,^11^,^12^. Furthermore, the synthesis of CH_3_NH_3_PbI_3_ (MAPbI_3_) goes through a simple process, by mixing PbI_2_ and MAI precursors^6^. In 2012, the superior performance via MAPbI_3_ synthesis with PbCl_2_ and MAI precursors was introduced by Snaith’s group, and property analyses were carried out by many groups^13^,^14^,^15^,^16^. Since then, researchers widely studied the chlorine effect, and concluded that chlorine enhances the morphology of perovskite films^16^,^17^,^18^,^19^,^20^. Even though the chlorine effect is suggested by many research groups, understanding the mechanisms on the synthesis is still required to be elucidated.

Architectural challenges are widely studied due to the ambipolar behavior^21^,^22^,^23^ of perovskites. Among them, the highest efficiency of 22.10%^7^ has been achieved with mesoporous structure, and mesoporous layer allows the additional light trapping effect^24^,^25^. In the mesoscopic structure, there are two major MAPbI_3_ deposition methods. The one-step solution deposition generally uses a mixture solution of PbI_2_ and MAI^16^, and the sequential deposition is carried out by pre-depositing the PbI_2_ film, followed by dipping it into an MAI-dissolved iso-propanol solution to form the MAPbI_3_ film^26^. Among them, the one-step solution deposition is highly beneficial in that this process is quite simple and time-saving. However, the sequential deposition is reported with a higher PCE than that of the one-step deposition^27^,^28^,^29^ due to the enhanced pore filling through the mesoporous TiO_2_ (mp-TiO_2_). Although the sequential deposition guarantees a high PCE, a comparative disadvantage in the sequential deposition is that it is a long-time process, since it goes through multiple steps to fabricate the perovskite film^26^.

In this article, we have demonstrated a straightforward diffusion-controlled synthesis approach by replacing the conventional MAI-dissolved iso-propanol solution with a MAI-dissolved ethanol solution, which enhanced the crystallinity, boosted the perovskite transformation, and minimized impurities. Moreover, we have detected intermediate phases when the PbCl_2_ precursor transforms into MAPbI_3_, and engineered the MAPbI_3_ deposition procedure by artificially mixing those intermediates as deposition precursors. This novel approach allowed superior surface morphology and crystallinity with enhanced conversion kinetics of MAPbI_3_, yielding an initial PCE of 11.23% and notable stability exhibiting 10.14% PCE after 30 days under ambient conditions.

## Results

### Ethanol Conversion

**Figure 1:**
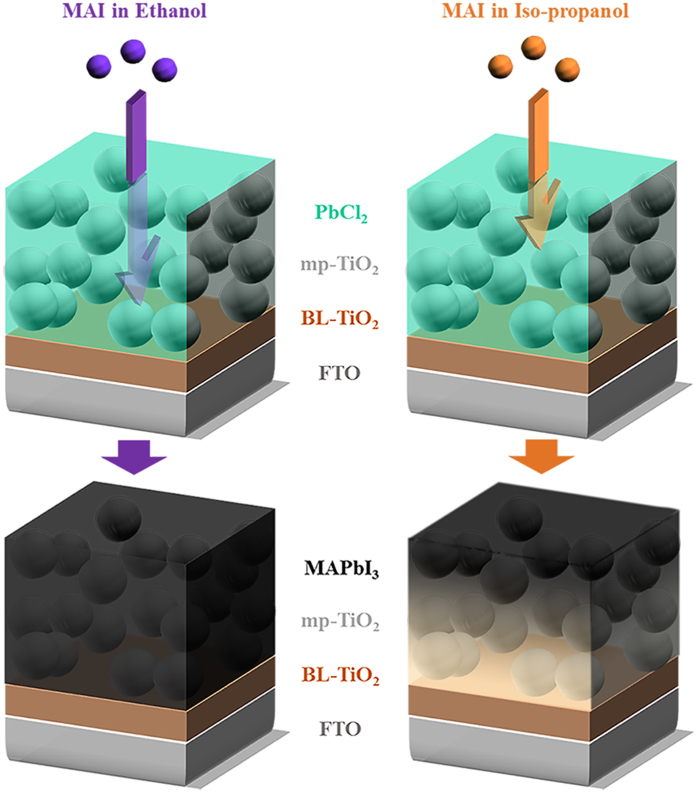
Schematic illustration of the MAI in ethanol or iso-propanol diffusing into the PbCl_2_-wetted mesoporous-TiO_2_ (mp-TiO_2_). The blocking-layer is coated on SnO_2_:F (FTO).

**Figure 2:**
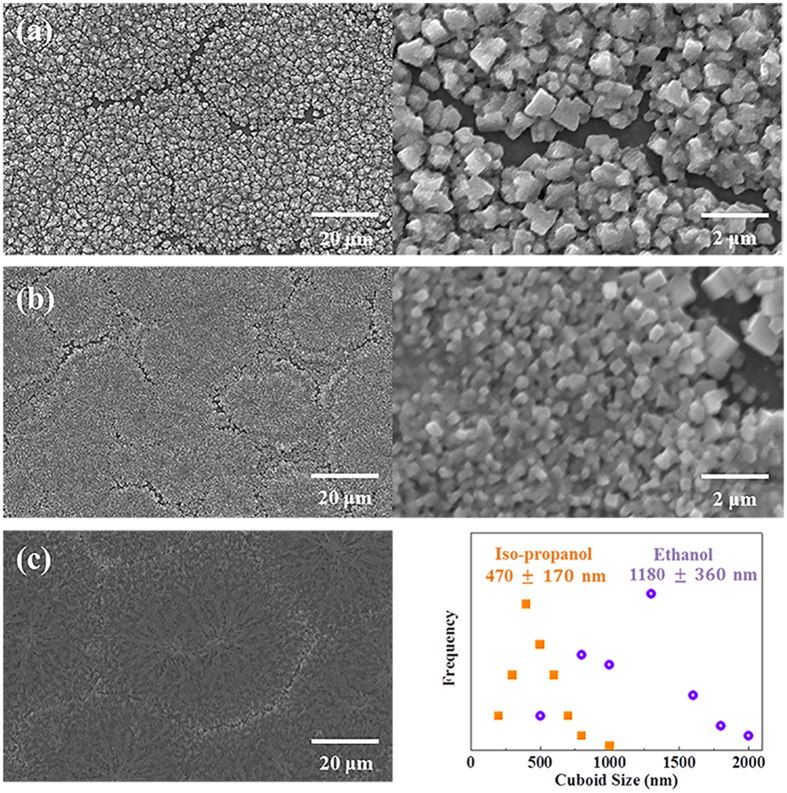
Solvent effects on MAPbI_3_ using ethanol or isoporpanol. (**a**) SEM images of MAPbI_3_ using ethanol, (**b**) MAPbI_3_ using iso-propanol, and (**c**) PbCl_2_ on mp-TiO_2_. The average cuboid size is shown for MAPbI_3_ by ethanol or iso-propanol.

Sequential deposition is one of the most preferable methods for perovskite fabrication due to the high PCE. One major problem, however, is the long fabrication time by multiple fabrication steps^26^. To reduce the fabrication time in the sequential deposition process, boosting the formation kinetics of MAPbI_3_ using MAI with PbCl_2_ precursors is required. Therefore, a low viscous solvent and larger concentration of MAI are necessary for effective diffusion of MAI into the PbCl_2_ layer. In general, conventional dipping solution uses 10 mg mL^−1^ of MAI in iso-propanol^26^, and a high concentration of MAI in solvent reduces both cuboid sizes and PCEs^30^. Thus, finding an alternative solvent is necessary for the viscosity and diffusion aspects. Figure 1 schematically illustrates the movements of ionized MAI into the PbCl_2_ film with ethanol (20 mg mL^−1^) and iso-propanol (10 mg mL^−1^), where the conversion kinetics of PbCl_2_ into MAPbI_3_ for each solvent is quite different even with the optical images (Supplementary Fig. 1, fast conversion kinetics with MAI/ethanol). The extent of the reaction was easily estimated by color changes (*E*_*g*_ of MAPbI_3_ ≈ 1.55 eV). However, less viscous methanol was not effective due to the dissolution of MAPbI_3_ (Supplementary Fig. 2)^26^. The fabricated perovskite film with the same concentration (20 mg mL^−1^) for the iso-propanol solution results in rather small cuboid sizes (~80 nm) with a low PCE of 2.08% in the solar cell performance, as shown in Supplementary Fig. 3. The cuboid size of MAPbI_3_ with an ethanol solution is also distinguishable from that with iso-propanol, as shown in the scanning electron microscopy (SEM) (Fig. 2a,b). The PbCl_2_-deposited film and cuboid-size distributions are plotted in Fig. 2c, and the synthesized perovskite with twice-large cuboid sizes (~1180 nm) through an ethanol conversion is expected to produce higher carrier mobilities^31^.

**Figure 3:**
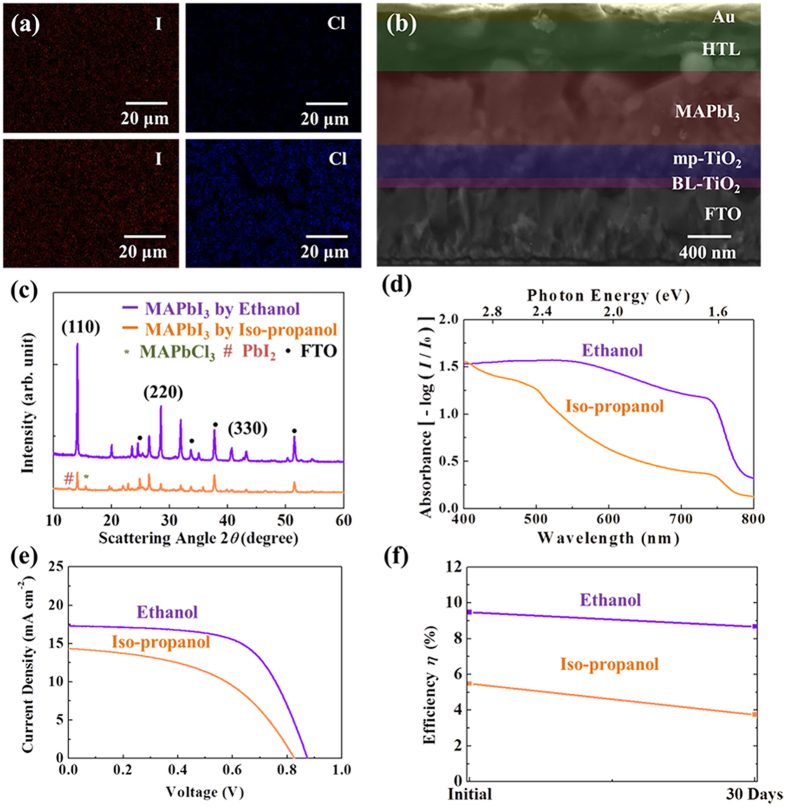
The dependence of MAPbI_3_ perovsktie solar cells using the MAI in an ethanol or iso-propanol solution. (**a**) EDS mapping for iodine and chlorine in the synthesized MAPbI_3_ with an ethanol (top) or iso-propanol (bottom) solution. (**b**) Cross-sectional SEM image of a MAPbI_3_ perovskite solar cell from the MAI/ethanol solution. (**c**) X-ray diffraction of MAPbI_3_. (**d**) Absorption spectra for the synthesized MAPbI_3_. (**e**) *J-V* characteristics of the MAPbI_3_ perovskite solar cells. (**f**) Degradation of solar cells synthesized by ethanol or iso-propanol for the as-fabricated cells and cells after 30 days.

Figure 3 illustrates qualitative analysis of the perovskite formation with ethanol or iso-propano. The energy disperse x-ray spectroscopy (EDS) was conducted to identify the chlorine concentration (Fig. 3a). MAPbI_3_ converted from an ethanol solution contains lower Cl than that of iso-propanol-synthesized MAPbI_3_ since unreacted PbCl_2_ or partially-reacted MAPbCl_3_ remains in the film. Still, ethanol-based MAPbI_3_ shows some chlorine content, and we believe that this is caused by MACl which is a co-product during the MAPbI_3_ synthesis^16^,^17^,^18^. Actually, there is a possibility that chlorine is doped in the perovskite structure (MAPbI_3-x_Cl_x_) as reported by several groups^14^,^15^,^16^. However, the small quantity of chlorine in the perovskite structure is difficult to be evaluated, and the remaining chlorine may form other products^17^,^19^. Cross-sectional SEM image of MAPbI_3_ perovskite solar cell by ethanol exhibits uniform film structures, as shown in Fig. 3b. For further understanding of impurities, X-ray diffraction (XRD) scans were compared, and an ethanol-based MAPbI_3_ shows clear (110), (220) and (330) peaks (Fig. 3c). On the contrary, the conversion with iso-propanol produced impurity peaks of PbI_2_ and MAPbCl_3_ (Fig. 3c), indicating incomplete reaction. To explain the effect of ethanol on the crystallinity, we have additionally confirmed that the longer dipping time increases the crystallinity of MAPbI_3_ (Supplementary Fig. 4) eVen when the reaction was completed. Together with the optical observation in Supplementary Fig. 1, it can be said that MAI/ethanol-converted MAPbI_3_ completes the reaction faster with better crystallinity, compared to that of iso-propanol for the same dipping time. Furthermore, the light absorption from the synthesized perovskite is clearly different between ethanol and iso-propanol (Fig. 3d). The enhanced absorption at approximately 800 nm by ethanol is due to the superior purity of MAPbI_3_ (*E*_*g*_ ≈ 1.55 eV), while partially-reacted MAPbI3 by iso-propanol contains high-bandgap impurities, such as PbI2 (*E*_*g*_ ≈ 2.36 eV)^32^ and MAPbCl_3_ (*E*_*g*_ ≈ 3.17 eV)^33^. Therefore, the overall PCE is greatly improved from 5.86% to 9.51% (Fig. 3e) with much better stability (Fig. 3f), and both methods performed high reproducibility (Supplementary Fig. 5 and Supplementary Table 1). After 30 days, the PCE decreased from 9.51% to 8.53% and 5.86% to 3.75%, respectively, for the ethanol and iso-propanol solution (Table 1). The improved crystallinity and enlarged grain of MAPbI_3_ by ethanol surely prevents possible air penetration through various grain boundaries, leading to stability enhancement. The half-lifetime of the MAPbI_3_ perovskite solar cell (degradation details in Supplementary Fig. 6) was estimated to be ~150 and ~40 days, respectively, for ethanol and iso-propanol.

**Figure 4:**
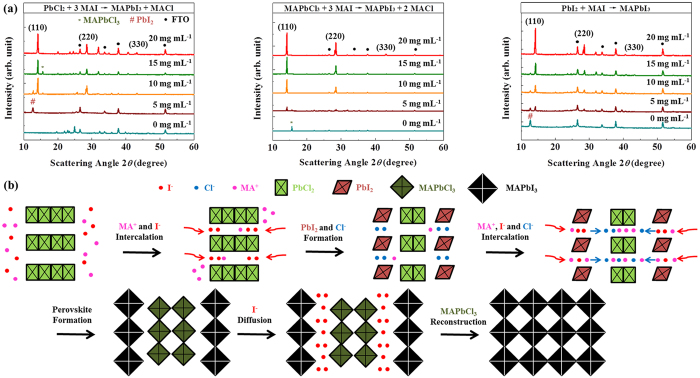
Reaction intermediates and mechanism analysis by X-ray diffraction measurements. (**a**) Reaction 1 (left), reaction 4 (middle), and PbI_2_ precursor (right) with various concentrations of MAI in an ethanol solution. (**b**) Mechanisms of reaction 1 (PbCl_2_ + 3 MAI → MAPbI_3_ + 2 MACl). The MAI and ethanol diffuse into the outer PbCl_2_ layer, which converts PbCl_2_ into PbI_2_ and MACl. Additional MAI from the solution converts the synthesized outer PbI_2_ into MAPbI_3_, and MACl reacts with the inner PbCl_2_ transforming into MAPbCl_3_. The synthesized MAPbCl_3_ ion-exchanges with I^−^ for the full MAPbI_3_ and MACl to complete the reaction.

To identify the reaction mechanisms, intermediate phases during the perovskite formation were investigated by the concentration variations of MAI in ethanol. With a low concentration of MAI/ethanol (Fig. 4a), PbCl_2_ partially reacts into PbI_2_ (5 mg mL^−1^). The chlorine in PbCl_2_ ion-exchanges with iodine in MAI to form PbI_2_, and the dissociated MA^+^ and Cl^−^ from outer PbCl_2_ intercalate into the inner PbCl_2_ layer, transforming to the MAPbCl_3_ phase (15 mg mL^−1^ of MAI/ethanol). The intermediate PbI_2_ reacts with MAI directly to form MAPbI_3_ by intercalating MAI in the layered PbI_2_, while MAPbCl_3_ will ion-exchange with I^−^ and reconstructs to the final MAPbI_3_, as schemed in Fig. 4b (20 mg mL^−1^ of MAI/ethanol). The whole reaction occurs through the following steps:


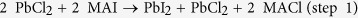



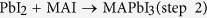



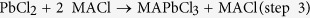



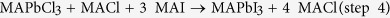



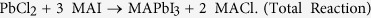


The PbI_2_ phase converts into MAPbI_3_ earlier than the formation of MAPbCl_3_, as shown by the X-ray diffraction of MAPbI_3_ vs. MAPbCl_3_ phases for MAI concentrations of 10 and 15 mg mL^−1^ (Fig. 4a). We have also compared the perovskite formation from MAPbCl_3_ (MAPbCl_3_ + 3 MAI → MAPbI_3_ + 3 MACl) with PbI_2_ (PbI_2_ + MAI → MAPbI_3_) (respectively, in the middle and right of Fig. 4a), and found that both have resulted in no intermediate phases. Synthesizing fully-converted MAPbI_3_ from PbCl_2_ requires both intercalation and reconstruction steps, while the idea of reconstruction from MAPbCl_3_ to MAPbI_3_ was investigated in the previous report^34^. Therefore, we intuitively conclude that the recrystallization of intermediates both inside and on top of the mp-TiO_2_ film can enhance the coverage morphology, nanostructures, and crystallinity^16^,^17^,^18^ of MAPbI_3_ by multiple crystal-alignment steps. Moreover, ethanol conversion increases the kinetics of the reaction steps, and produces improved MAPbI_3_ film, compared to the conversion with iso-propanol.

### Reaction Mechanism Engineering

**Figure 5:**
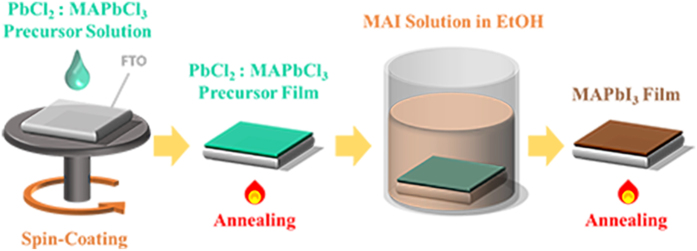
Schematic illustration of the experimental procedure. Various ratios of PbCl_2_: MAPbCl_3_ were initially deposited on the substrate, and then the MAI-dissolved ethanol solution was utilized to synthesize MAPbI_3_.

To understand the phase-formation paths of MAPbI_3_ from PbCl_2_, we came up with an idea to further optimize MAPbI_3_ by utilizing the identified intermediate phases. While the direct conversion of MAPbCl_3_ to MAPbI_3_ can drastically reduce the reaction time, the repetition of crystallization from PbCl_2_ to MAPbI_3_ will enhance the surface morphology. Even though PbI_2_ appears during the transformation of the PbCl_2_ precursor to MAPbI_3_, and increases the reaction kinetics, the MAPbCl_3_ precursor is more reactive than the PbI_2_ precursor to synthesize MAPbI_3_ (middle and right graphs in Fig. 4a) where the MAPbCl_3_ precursor is likely to transform with lower MAI concentration than that of the PbI_2_ precursor. Also, the chlorine-based precursor should be preferred considering the positive effects of chlorine on the MAPbI_3_ perovskite solar cells^16^,^17^,^18^,^19^,^20^. Therefore, a straightforward direction is rendered by mixing PbCl_2_ and MAPbCl_3_ in several different ratios of 3:0 (reaction 1), 2:1 (reaction 2), 1:2 (reaction 3), and 0:3 (reaction 4), to optimize the reaction time with smooth surface morphology (Table 2 and Fig. 5).

**Figure 6:**
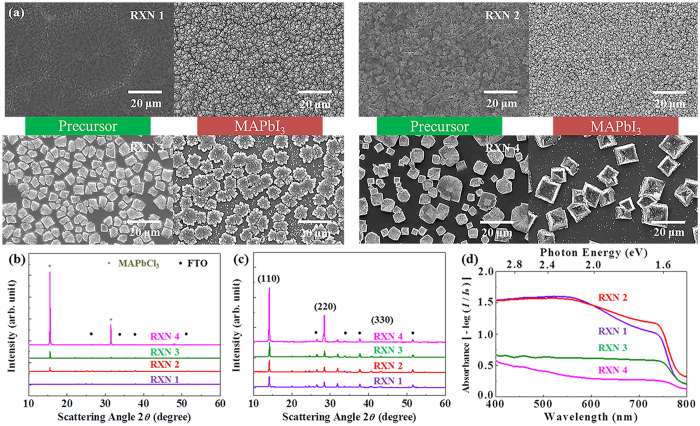
The film quality dependence of MAPbI_3_ for the reactions 1, 2, 3, and 4. (**a**) SEM images of the precursor and MAPbI_3_, (**b,c**) X-ray diffraction for the precursor and MAPbI_3_, and (**d**) absorption spectra of MAPbI_3_ by reactions 1, 2, 3, and 4.

**Figure 7:**
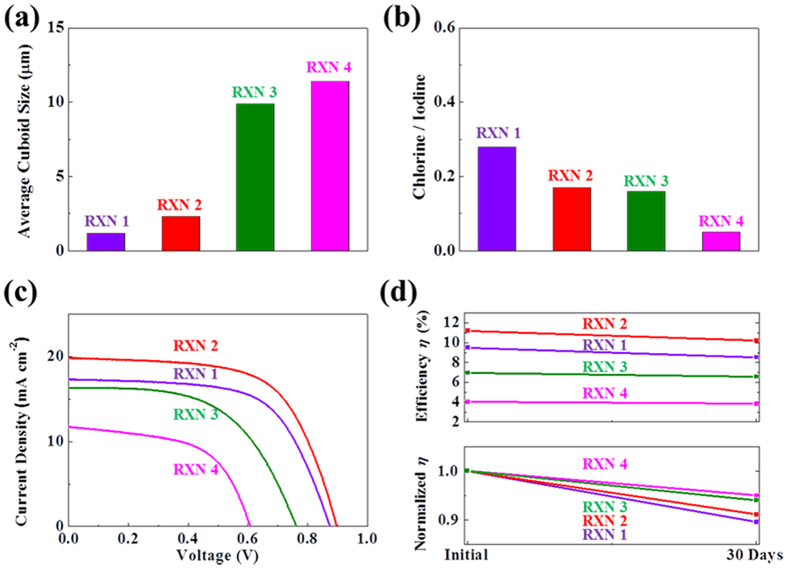
The purity and deivce performance analysis for the reactions 1, 2, 3, and 4. (**a**) Average cuboid size, (**b**) EDS for the comparative chlorine contents with iodine contents, (**c**) *J-V* curves, and (**d**) the efficiency degradation for the as-fabricated cells and cells after 30 days.

As expected, we observed the morphology changes by synthesizing MAPbI_3_ films from precursors with different ratios of PbCl_2_ and MAPbCl_3_ through SEM, as shown in Fig. 6a. Perovskite films synthesized by reaction 2 exhibits clearly improved coverage with ~2320-nm-sized cuboids. Furthermore, addition of MAPbCl_3_ (reaction 3 and 4) deteriorates the coverage, but increases the cuboid size. When a larger amount of the MAPbCl_3_ precursor is added through reactions, the crystallinity of MAPbI_3_ is enhanced significantly, as shown by XRD (Fig. 6b,c). The enhanced crystallinity can be explained by the extent of reaction, and the facilely-transformed perovskite is likely to have high crystallinity even with the same dipping time (Supplementary Fig. 4). These observations are also consistent with the optical variations during the MAPbI_3_ formation (Supplementary Fig. 7). It should be noted that the coverages for the reactions 1, 2, 3, and 4 are different. However, conversions at ~10 s are distinct between reactions 1 (PbCl_2_ precursor) and 4 (MAPbCl_3_ precursor). The light absorption in Fig. 6d indicates that the absorption is more influenced by the coverage rather than the crystallinity and cuboid size of MAPbI_3_. The maximum coverage in reaction 2 reached the highest absorption, and the minimum coverage with reaction 4 yielded the lowest absorption. As a material perspective, MAPbI_3_ synthesis by reaction 4 is supposed to show excellent properties due to the high crystallinity and cuboid sizes, as plotted in Fig. 7a. Moreover, EDS was additionally measured to identify the comparative chlorine contents with iodine, which is plotted in Fig. 7b for each reaction, indicating that reaction 1 obtained the highest, and reaction 4 occupied the lowest concentration of chlorine. This chlorine tendency suggests that a co-product of MACl is minimized through the addition of MAPbCl_3_. (It should be noted that the EDS technique may not reflect the accurate chlorine concentration due to the coverage difference of each reaction.) It is possible that the detected chlorine through EDS is from the MACl phase or other products^16^,^17^,^18^. Moreover, excessive chlorine may lead to impurities, and deteriorate the device performance. Therefore, high crystallinity and low impurity of MAPbI_3_ are highly beneficial in the carrier mobilities, but recombination of carriers arising from poor coverage^31^ is another factor that we should be aware of.

To understand the effects of crystallinity and coverage on the photovoltaic performance and stability, *J-V* curves are measured for 30 days under ambient conditions (Supplementary Fig. 6, and Table 3). In Fig. 7c, *J-V* curves were shown for the performance of the solar cells from each reaction, and the highly covered perovskite film from reaction 2 achieved the highest PCE of 11.23%. The lowest PCE of 4.03% was obtained by reaction 4, and these results indicate that the initial PCE is highly dependent on the perovskite coverage, which plays crucial roles in the carrier recombination. In contrast, MAPbI_3_ synthesized by reaction 4 was distinctively stable after 30 days. The stability is well correlated with the crystallinity and grain size, apparent from the normalized PCE in Fig. 7d, confirming the reduced decomposition behavior from the low-defect perovskite. All of the solar cells with different experimental conditions performed high reproducibility (Supplementary Fig. 5 and Supplementary Table 1). From the reactions 1, 2, 3, and 4, the half-lifetimes of MAPbI_3_ perovskite solar cells (degradation details in Supplementary Fig. 6) are estimated to be ~150, ~160, ~270, and ~300 days.

## Discussion

Fast conversion can have positive influences on the device performance and stability by producing highly-crystalline MAPbI_3_ perovskite. Therefore, we have controlled the diffusion reaction of MAI and PbCl_2_ with ethanol to boost the perovskite transformations, leading to phase-pure and highly-crystalline perovskite films. Since PbCl_2_ goes through several intermediate phases during the formation of MAPbI_3_, we utilized the intermediates by mixing them with a conventional PbCl_2_ precursor to boost the conversion kinetics and performance of the resulting solar cell. Thereby, the optimized crystallinity and coverage yielded a PCE of 11.23% with PbCl_2_: MAPbCl_3_ = 2:1 (reaction 2). Although the precursor with 100% MAPbCl_3_ (reaction 4) resulted in the fastest transformations of MAPbI_3_ and the most stable solar cell performance, poor coverage lowered the PCE of the device. Therefore, compact coverage of the perovskite with super-sized cuboids expects to achieve further enhanced performance and stability of the MAPbI_3_ perovskite solar cell. At this point, investigation of hysteresis still remains as a future work.

## Methods

### Perovskite Solar Cell Fabrication

Fluorine-doped tin oxide substrate (FTO, TEC 8: Pilkington) was cleaned by sonication in Mucasol (Aldrich), ethanol (DEAJUNG), and DI water for 30 min sequentially. 50 nm of compact the TiO_2_ blocking layer was deposited by spin-coating the mixture solution of 0.15 mM titanium diisopropoxide bis(acetylacetonate) (Aldrich) and 1-butanol (75.0 wt. % in iso-propanol, Aldrich) in 2500 rpm for 20 s followed by heating at 125 °C for 5 min in an air oven. The same step was repeated with 0.3 mM concentration and the substrate was annealed at 500 °C for 30 min. After the TiO_2_ blocking layer was ready, TiO_2_ pastes (ENB Korea) with 20 nm-sized nanoparticles were mixed with terpineol (Aldrich) in 1:2 ratio, followed by spin-coating at 4000 rpm for 30 s, yielding a ~350 nm thickness of the mp-TiO_2_ layer. For the perovskite synthesis, MAI was first synthesized by following literature method^26^. 1.5 M PbCl_2_ (Aldrich) was diluted in dimethyl sulfoxide (DMSO, Aldrich), and then MACl (Aldrich) was added with different concentrations (0, 0.5, 1.0, and 1.5 M) to synthesize the reaction 1, 2, 3, and 4 precursors where the reaction 1, 2, and 3 precursors contain both MAPbCl_3_ and PbCl_2_ phases, whereas the reaction 4 precursor contains only MAPbCl_3_ (MAPbCl_3_ forms by 1:1 molar stoichiometric ratios of MACl and PbCl_2_). After the preparation of precursor mixture solutions, the solution was preheated at 100 °C and the substrate was preheated at 150 °C, then the solution was spin-coated at 2000 rpm for 5 s, followed by 6000 rpm for 5 s. The film was annealed at 150 °C for 30 min, and cooled down in an ambient condition. After the film was cooled down, it was dipped into 20 mg mL^−1^ of MAI in an anhydrous ethanol solution (Daejung) for 20 min under ambient conditions (25 °C and 55% humidity) and annealed at 100 °C for 30 min. The hole transport solution was prepared by mixing 72.3 mg mL^−1^ of spiro-OMeTAD (Merck) in chlorobenzene (Aldrich) with 28.8 μL of *tert*-butylpyridine (Aldrich) and a 17.5 μL solution of 520 mg of lithium *bis*(trifluoromethylsyfonyl)imide salt (Aldrich) in 1 mL acetonitrile (Aldrich) was spin-coated at 3000 rpm for 45 s. Finally, 100 nm thickness of an Au electrode was then thermally evaporated.

### Device Characterizations

The morphologies of MAPbI_3_ perovskite films were analyzed using scanning electron microscope (Normal-SEM, JSM-6360: Hitachi). The chlorine compositions and distribution were examined using energy-dispersive X-ray spectroscopy (EDS, ISIS-300: Oxford Instruments). The phases of the synthesized samples were characterized by X-ray diffraction (XRD, D8 Advance: Bruker). The photocurrent-voltage (*J–V*) curves of MAPbI_3_ perovskite solar cells were obtained with a potentiostat (CHI 608C: CH Instrumental Inc.) under AM 1.5 illumination at 100 mW cm^−2^ (K3000: McScience) with an active cell area of 0.09 cm^2^. The field-emission scanning electron microscope (FE-SEM, Merlin-Compact: Carl Zeiss) was used to observe the plan and cross-sectional views. The absorption spectra of the MAPbI_3_-deposited films were recorded on a UV-Vis spectrophotometer (Lambda 20: Perkin Elmer). Stability was measured every 5 days, and stored at 25 °C with 55% of humidity under dark conditions.

## Additional Information

**How to cite this article**: Kim, J. *et al.* Solvent and Intermediate Phase as Boosters for the Perovskite Transformation and Solar Cell Performance. *Sci. Rep.* **6**, 25648; doi: 10.1038/srep25648 (2016).

## Supplementary Material

Supplementary Information

## Figures and Tables

**Table 1:** Photovoltaic performance of MAPbI_3_ perovskite solar cells using an ethanol or iso-propanol solution (as-fabricated cells and cells after 30 days).

Reaction Condition		*V*_*oc*_(V)	*J*_*sc*_(mA/cm^2^)	*FF*	*η*(%)
Initial	Ethanol	0.87	17.3	0.63	9.51
	Iso-propanol	0.83	14.4	0.49	5.86
30 Days	Ethanol	0.84	16.4	0.63	8.53
	Iso-propanol	0.79	10.1	0.47	3.75

**Table 2:** Precursor ratio of PbCl_2_: MAPbCl_3_ for the reactions 1, 2, 3, and 4.

	Reaction Mechanism	Precursor Ratio (PbCl_2_: MAPbCl_3_)
RXN 1	PbCl_2_ + 3 MAI → MAPbI_3_ + 2 MACl	3:0
RXN 2	2 PbCl_2_ + MAPbCl_3_ + 9 MAI → 3 MAPbI_3_ + 7 MACl	2:1
RXN 3	PbCl_2_ + 2 MAPbCl_3_ + 9 MAI → 3 MAPbI_3_ + 8 MACl	1:2
RXN 4	MAPbCl_3_ + 3 MAI → MAPbI_3_ + 3 MACl	0:3

**Table 3:** Solar cell performance from the reactions 1, 2, 3, and 4 (as-fabricated cells and cells after 30 days).

Reaction Conditions		*V*_*oc*_(V)	*J*_*sc*_ (mA cm^−2^)	*FF*	*η*(%)
Initial	RXN 1	0.87	17.3	0.63	9.51
	RXN 2	0.90	19.8	0.63	11.23
	RXN 3	0.76	16.3	0.56	6.94
	RXN 4	0.61	11.7	0.57	4.03
30 Days	RXN 1	0.84	16.4	0.62	8.53
	RXN 2	0.89	17.8	0.64	10.14
	RXN 3	0.73	15.2	0.59	6.55
	RXN 4	0.61	11.7	0.52	3.83
