# Supplementary material for: Solvent and Intermediate Phase as Boosters for the Perovskite Transformation and Solar Cell Performance
Source: Sci Rep. 2016 May 9;6:25648. doi: 10.1038/srep25648 (PMC4860713; doi:10.1038/srep25648)
Supplement: Supplementary Information [file srep25648-s1.pdf]

Solvent and Intermediate Phase as Boosters for the Perovskite Transformation and Solar Cell Performance

*Jinhyun Kim, Taehyun Hwang, Sangheon Lee, Byungho Lee, Jaewon Kim, Gil Su Jang, Seunghoon Nam, and Byungwoo Park**

WCU Hybrid Materials Program, Department of Materials Science and Engineering,

Research Institute of Advanced Materials,

Seoul National University, Seoul 08826, Korea

Device	Iso-propanol	RXN 1	RXN 2	RXN 3	RNX 4
1	5.89%	8.87%	9.95%	6.95%	4.40%
2	5.87%	9.51%	11.00%	7.32%	4.03%
3	5.53%	9.43%	10.12%	7.31%	4.16%
4	5.84%	8.07%	11.19%	6.29%	3.76%
5	5.91%	8.76%	10.10%	6.87%	4.23%
6	5.31%	8.50%	10.10%	6.94%	4.24%
7	5.23%	8.55%	11.24%	6.44%	3.80%
8	5.15%	9.09%	10.00%	6.51%	3.83%
9	6.30%	8.55%	12.30%	6.67%	3.62%
10	5.86%	9.09%	11.80%	7.29%	4.58%

Supplementary Table 1. Power conversion efficiencies of $MAPbI_3$ perovskite solar cells from the reactions 1, 2, 3, 4, and iso-propanol.

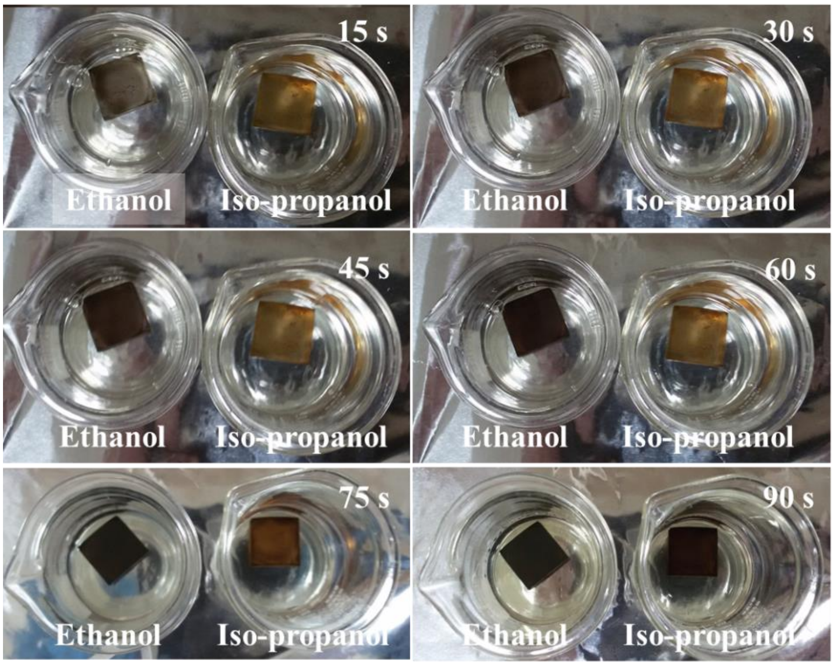

Supplementary Figure 1. Time-dependent optical variation for the $PbCl_2$ films in the MAI/ethanol or MAI/iso-propanol solution.

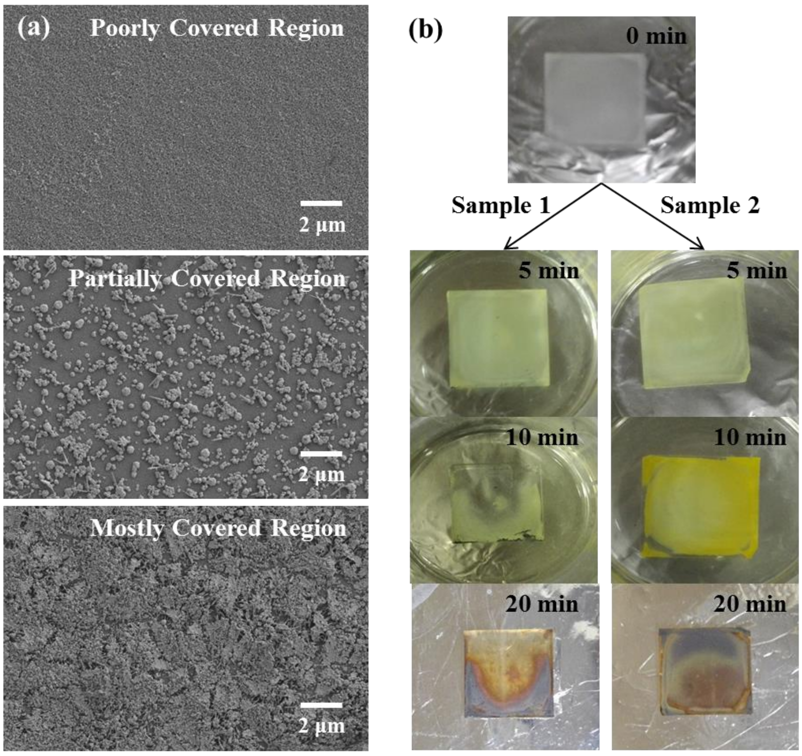

Supplementary Figure 2. $MAPbI_3$ conversion using a methanol solvent: (a) SEM images for the $MAPbI_3$ film in different regions (20-min dipping sample), and (b) optical observations in every 5 min.

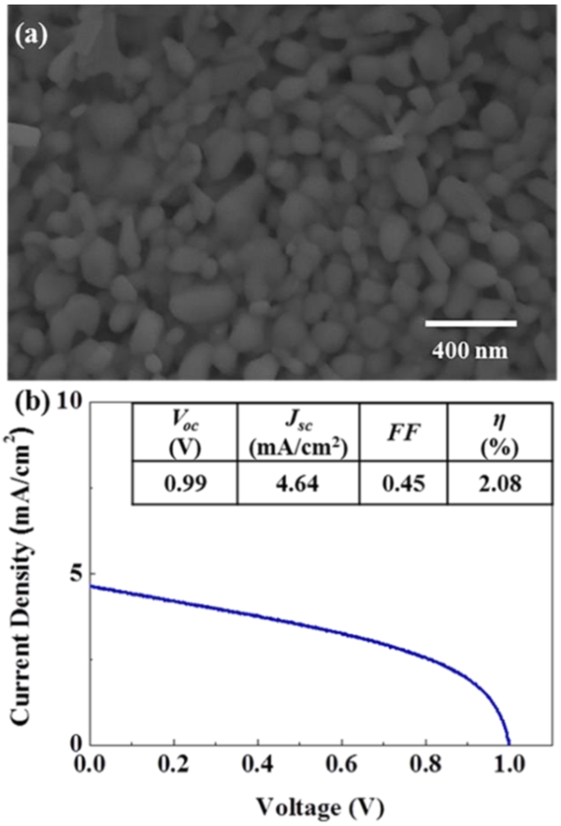

Supplementary Figure 3. SEM images of (**a**) $MAPbI_3$ and (**b**) *J-V* curve from the 20-mg/mL MAI/iso-propanol solution.

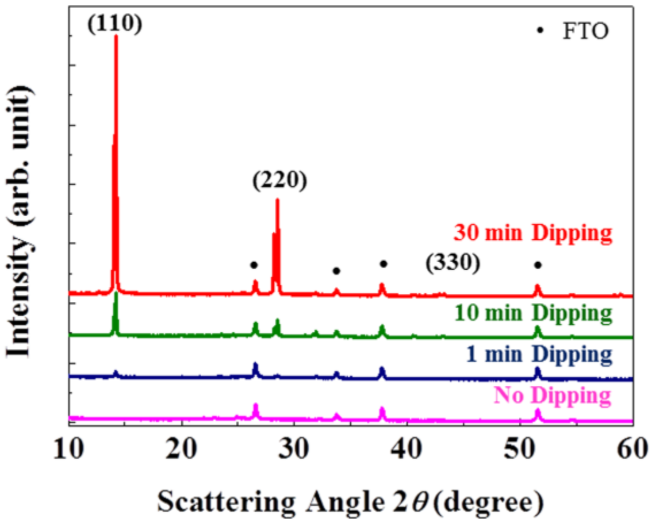

Supplementary Figure 4. X-ray diffraction from various MAI/ethanol dipping times on the $PbCl_2$ film.

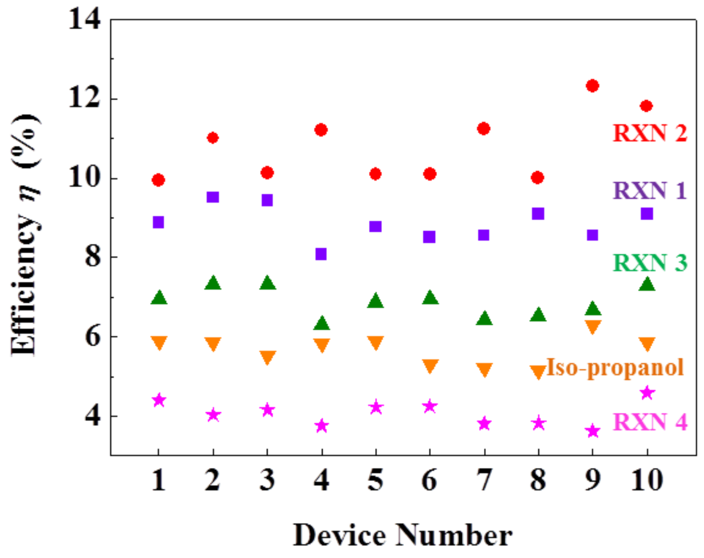

Supplementary Figure 5. Device reproducibility of $MAPbI_3$ perovskite solar cells from the reactions 1, 2, 3, 4, and iso-propanol.

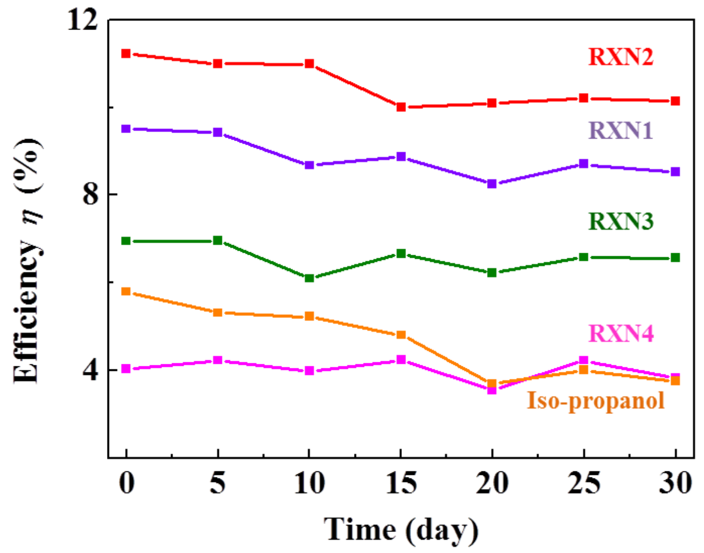

Supplementary Figure 6. Stability of $MAPbI_3$ perovskite solar cells from the reactions 1, 2, 3, and 4 for 30 days. The corresponding data by iso-propanol (RXN 1) are also shown.

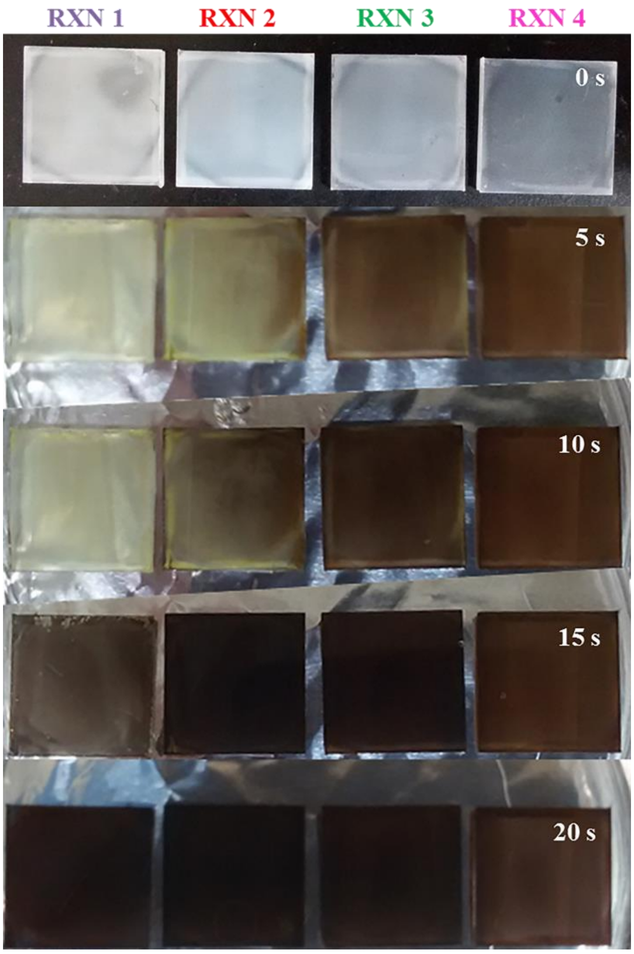

Supplementary Figure 7. Time-dependent optical variation for the reactions 1, 2, 3, and 4 in a MAI/ethanol solution.
